# Supplementary material for: Novel Driver Strength Index highlights important cancer genes in TCGA PanCanAtlas patients
Source: PeerJ. 2022 Aug 11;10:e13860. doi: 10.7717/peerj.13860 (PMC9375969; doi:10.7717/peerj.13860)
Supplement: Supplemental Information 2 [file peerj-10-13860-s002.zip › COHORTS/genes plots/detailed/2021_8_16_14_9_distribution_events_detailed_1.pdf]

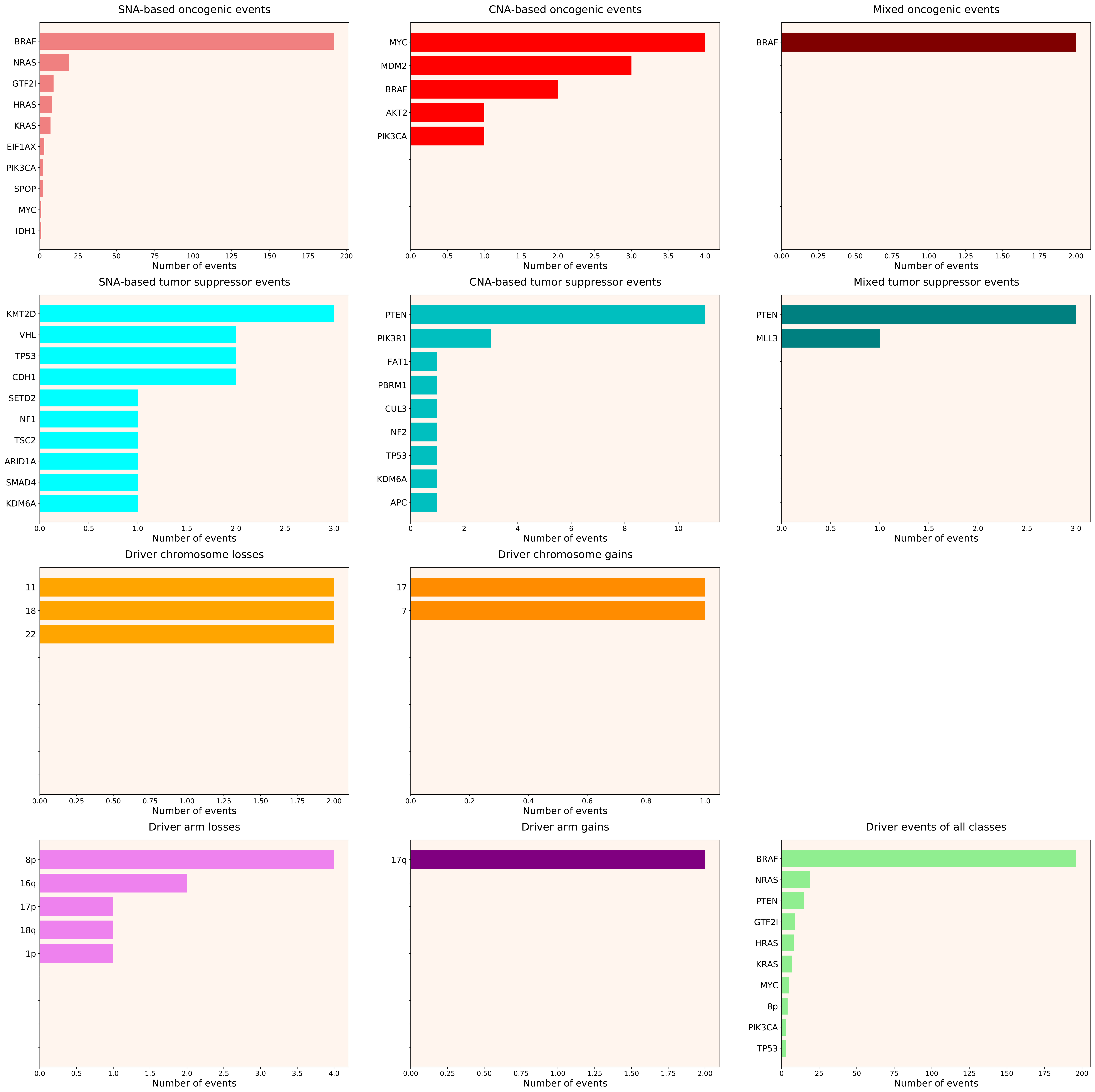

SNA-based oncogenic events
BRAF
NRAS
GTF2I
HRAS
KRAS
EIF1AX
PIK3CA
SPOP
MYC
IDH1
Number of events
CNA-based oncogenic events
MYC
MDM2
BRAF
AKT2
PIK3CA
Number of events
Mixed oncogenic events
BRAF
Number of events
SNA-based tumor suppressor events
KMT2D
VHL
TP53
CDH1
SETD2
NF1
TSC2
ARID1A
SMAD4
KDM6A
Number of events
CNA-based tumor suppressor events
PTEN
PIK3R1
FAT1
PBRM1
CUL3
NF2
TP53
KDM6A
APC
Number of events
Mixed tumor suppressor events
PTEN
MLL3
Number of events
Driver chromosome losses
11
18
22
Number of events
Driver chromosome gains
17
7
Number of events
Driver arm losses
8p
16q
17p
18q
1p
Number of events
Driver arm gains
17q
Number of events
Driver events of all classes
BRAF
NRAS
PTEN
GTF2I
HRAS
KRAS
MYC
8p
PIK3CA
TP53
Number of events
